# The association between anterior nares and nasopharyngeal microbiota in infants hospitalized for bronchiolitis

**DOI:** 10.1186/s40168-017-0385-0

**Published:** 2018-01-03

**Authors:** Pamela N. Luna, Kohei Hasegawa, Nadim J. Ajami, Janice A. Espinola, David M. Henke, Joseph F. Petrosino, Pedro A. Piedra, Ashley F. Sullivan, Carlos A. Camargo, Chad A. Shaw, Jonathan M. Mansbach

**Affiliations:** 1 Department of Statistics, Rice University, Houston, TX USA; 2 Department of Emergency Medicine, Massachusetts General Hospital, Harvard Medical School, Boston, MA USA; 3 Alkek Center for Metagenomics and Microbiome Research, Department of Molecular Virology and Microbiology, Baylor College of Medicine, Houston, TX USA; 4 Department of Molecular and Human Genetics MS 225, Baylor College of Medicine, Houston, TX 77030 USA; 5 Department of Molecular Virology and Microbiology and Pediatrics, Baylor College of Medicine, Houston, TX USA; 6 Department of Medicine, Boston Children’s Hospital, Boston, MA USA

**Keywords:** Microbiota, Bronchiolitis, Asthma, Nasopharynx, Anterior nares

## Abstract

**Background:**

The airway microbiome is a subject of great interest for the study of respiratory disease. Anterior nare samples are more accessible than samples from deeper within the nasopharynx. However, the correlation between the microbiota found in the anterior nares and the microbiota found within the nasopharynx is unknown. We assessed the anterior nares and nasopharyngeal microbiota to determine (1) the relation of the microbiota from these two upper airway sites and (2) if associations were maintained between the microbiota from these two sites and two bronchiolitis severity outcomes.

**Results:**

Among 815 infants hospitalized at 17 US centers for bronchiolitis with optimal 16S rRNA gene sequence reads from both nasal swab and nasopharyngeal aspirate samples, there were strong intra-individual correlations in the microbial communities between the two sample types, especially relating to *Haemophilus* and *Moraxella* genera*.* By contrast, we found a high abundance of *Staphylococcus* genus in the nasal swabs—a pattern not found in the nasopharyngeal samples and not informative when predicting the dominant nasopharyngeal genera. While these disparities may have been due to sample processing differences (i.e., nasal swabs were mailed at ambient temperature to emulate processing of future parent collected swabs while nasopharyngeal aspirates were mailed on dry ice), a previously reported association between *Haemophilus*-dominant nasopharyngeal microbiota and the increased severity of bronchiolitis was replicated utilizing the nasal swab microbiota and the same outcome measures: intensive care use (adjusted OR 6.43; 95% CI 2.25–20.51; *P* < 0.001) and hospital length-of-stay (adjusted OR 4.31; 95% CI, 1.73–11.11; *P* = 0.002). Additionally, *Moraxella*-dominant nasopharyngeal microbiota was previously identified as protective against intensive care use, a result that was replicated when analyzing the nasal swab microbiota (adjusted OR 0.30; 95% CI, 0.11–0.64; *P* = 0.01).

**Conclusions:**

While the microbiota of the anterior nares and the nasopharynx are distinct, there is considerable overlap between the bacterial community compositions from these two anatomic sites. Despite processing differences between the samples, these results indicate that microbiota severity associations from the nasopharynx are recapitulated in the anterior nares, suggesting that nasal swab samples not only are effective sample types, but also can be used to detect microbial risk markers.

**Electronic supplementary material:**

The online version of this article (10.1186/s40168-017-0385-0) contains supplementary material, which is available to authorized users.

## Background

The composition and function of the airway microbiota have been associated with respiratory conditions, such as pneumonia [1, 2], asthma [3–9], and chronic obstructive pulmonary disease [4, 10–12]. However, the airway is vast and extends from the nasal openings to the alveoli deep within the lungs. Ideally, samples for microbial analysis would be collected as close as possible to the cellular inflammatory responses contributing to the respiratory illness in question, which occur in the lower respiratory tract for bronchiolitis.

Unfortunately, the deeper segments of the airway (the lower respiratory tract) must be assessed through more invasive methods, such as bronchoscopy, which is not feasible for large-scale studies. Furthermore, easy sample collection is especially important for studies involving infants and young children. As a result, multiple studies in this young population have been conducted on the microbiome using nasal swabs (NSs) or nasal brush specimens, which are easier to collect and less invasive than samples from deeper within the airway [3, 6, 7, 13, 14].

Questions remain about the correlation between the microbiota within different microenvironments along the airway. Although prior studies have demonstrated strong correlations between upper and lower airway microbiology [15, 16] and virology [17], other data have suggested significant spatial variation in the composition and structure of the upper and lower airway microbiota [18, 19]. Additionally, there are emerging data about the correlation between the microenvironments in the upper airway [7, 20]. Yan et al. found in 12 healthy adults that the microbiota of the anterior nares was significantly different from that of the middle meatus and sphenoethmoidal recess [20]. Pérez-Losada et al. compared the microbiota of the inferior turbinate and nasopharynx among 30 children and adolescents with asthma [7] and found that these two regions had distinct microbial compositions. However, both of these studies were single-center studies with less than 40 participants. Moreover, while previous studies have examined compositional differences between microenvironments, no previous study has assessed if there are differences in the associations between the microbiota from different regions of the upper airway and a clinical outcome, particularly among young children and infants for whom less invasive sampling methods are imperative.

As part of the 35th Multicenter Airway Research Collaboration (MARC-35) study, site teams collected both NS and nasopharyngeal aspirate (NPA) samples from children hospitalized for bronchiolitis within 24 h of hospitalization [21]. Our two objectives in this secondary analysis of MARC-35 data were to (1) compare the microbial composition between the anterior nares and nasopharynx of infants hospitalized with bronchiolitis and (2) assess if associations between NPA microbial composition and bronchiolitis severity [21] would be replicated or enhanced using NS microbial data. We hypothesized that although systematic co-analysis of NS and NPA samples from MARC-35 participants would have shared and divergent microbial compositions, they would both be associated with severity of illness.

## Methods

### Study design

The 35th Multicenter Airway Research Collaboration (MARC-35) is a multicenter prospective cohort study of infants (age < 1 year) hospitalized for bronchiolitis. The study was coordinated by the Emergency Medicine Network (EMNet) [22]. MARC-35 was conducted at 17 sites across the USA during three consecutive bronchiolitis seasons (November 1 to April 30) from 2011 to 2014.

Participants consisted of infants diagnosed with bronchiolitis (as defined by the American Academy of Pediatrics) by the attending physician [23]. The exclusion criteria included previous enrollment in the study, consent to the study more than 24 h after hospitalization, transfer to a participating hospital more than 24 h after hospitalization, and known heart-lung disease, immunodeficiency, immune suppression, or gestational age less than 32 weeks. The institutional review board at each of the 17 participating hospitals approved the study, and patients were treated at the discretion of the attending physician.

### Data collection

Structured interviews of the parent or guardian were performed by site investigators to determine the demographic characteristics, medical and family history, and details of the acute illness for each patient. Additional clinical details were collected via emergency department and hospital inpatient chart reviews. Reviewers at the EMNet Coordinating Center reviewed all data and inquired about discrepancies and missing data with site investigators.

Trained site investigators collected NS and NPA samples using standardized protocols [24, 25]. The site investigators collected NS samples from the anterior nares within 24 h of hospitalization. Both nares were swabbed with a single nylon, pediatric FLOQSwab (Copan, Brescia, Italy). The NS samples were placed into a vial containing 2 mL of transport media (15% glycerol in Iscove’s media) and mailed to Massachusetts General Hospital (MGH) (Boston, MA) via US mail, where they were stored at − 80°C upon receipt. NS samples were then shipped on dry ice from MGH to Baylor College of Medicine (Houston, TX) via overnight mail, where they were again stored at − 80°C. The procedure used to collect the NS emulated future parent collected nasal swabs and shipping to ensure maximum comparability of all the nasal swab specimens collected in MARC-35.

For the NPA samples, all site teams used the same collection equipment (Medline Industries, Mundelein, IL) and also collected the samples within 24 h of hospitalization. For the collection, the child was placed supine; 1 mL of normal saline was instilled into one nare, and then an 8 French suction catheter was used to remove the mucus. This procedure was performed once on each nostril. After the sample collection from both nares, 2 mL of normal saline was suctioned through the catheter to clear the tubing. Immediately after collection, the NPA sample was added to the same transport medium as the NS in a 1:1 ratio and placed on ice. Within 1 h of collection, the NPA sample was refrigerated at 4 °C. Within 24 h of collection, the sample was transferred to a − 80 °C freezer until shipped on dry ice to Baylor College of Medicine (Houston, TX), where they were again stored at − 80°C.

### Microbiota community profiling

The composition of NS and NPA microbiota was characterized at the Alkek Center for Metagenomics and Microbiome Research (CMMR) at Baylor College of Medicine by sequencing the bacterial 16S rRNA gene V4 region on the Illumina MiSeq platform as described in the initial analysis of MARC-35 data. The NPA samples were additionally tested for 17 viral pathogens (e.g., rhinovirus, respiratory syncytial virus) using real-time polymerase chain reaction (PCR) assays [21]. Briefly, bacterial genomic DNA was extracted using MO BIO PowerSoil DNA Isolation Kit (MO BIO Laboratories). The 16S rDNA V4 region was amplified by PCR and sequenced in the MiSeq platform (Illumina) using the 2 × 250 bp paired-end protocol yielding pair-end reads that overlap almost completely. The primers used for amplification contain adapters for MiSeq sequencing and single-end barcodes allowing pooling and direct sequencing of PCR products [26]. Sequencing read pairs were demultiplexed based on the unique molecular barcodes, and reads were merged using USEARCH v7.0.1090 [27] allowing zero mismatches and a minimum overlap of 50 bases. Merged reads were trimmed at the first base with a Q5 quality score. We calculated the expected error after taking into account all Q-scores across all the bases of a read and the probability of an error occurring [28]. Additionally, a quality filter was applied to the resulting merged reads, and reads containing > 0.05 expected errors was discarded. Rarefaction curves of bacterial operational taxonomic units (OTUs) were constructed using sequence data for each sample to ensure coverage of the bacterial diversity present. Samples with suboptimal amounts of sequencing reads were re-sequenced to ensure that the majority of bacterial taxa were encompassed in our analyses. Positive and negative controls were included in the extraction, amplification, and sequencing processes together with the study samples for quality control and assurance purposes. The positive control consisted of a known and previously sequenced bacterial genome that is not expected to be found in the study samples. Negative controls were non-template controls composed of the reagents used in every process from sample extraction to sequencing. There was amplification on positive controls and no amplification on negative controls.

16S rRNA gene sequences were clustered into OTUs at a similarity cutoff value of 97% using the UPARSE algorithm [29]. OTUs were determined by mapping the centroids to the SILVA database [30] containing only the 16S V4 region to determine taxonomies. A custom script constructed a rarefied OTU table from the output files generated in the previous two steps for downstream analyses of alpha-diversity (e.g., Shannon index) and beta-diversity (e.g., weighted UniFrac distance matrix) [31, 32]. Shannon diversity index is a quantitative measure that takes into account not only richness but also the proportion of each bacteria (evenness) within the local community. The weighted UniFrac algorithm calculates the distance between microbial communities based on the phylogenetic relatedness of lineages and relative abundance in each sample.

### Statistical analyses

The relative abundance of each OTU was calculated for each sample, and the OTUs were combined at the genus level. For each sample type, the overall abundance of each genus was calculated by taking the sum of relative abundances by genus over all subjects. The union of the 10 most abundant genera for each site defined the overall 15 top genera for comparative analyses.

To determine whether there was a significant intra-individual correlation between the two upper airway sites, a permutation test was performed on the mean Spearman correlation for the NS and NPA sample pairs. Computing the Spearman correlation matrix of the top genera abundance for NS versus NPA assessed the intra-individual correlations by genera. Due to the compositional nature of microbiota data, Spearman correlation calculations can overestimate relationships in the data [33]. To determine the significance of the co-occurrence and mutual exclusion relationships between NS and NPA, a correlation network between the two sites was constructed using the approach of Faust et al. [34]. To construct the network, all genera that appeared in at least 10% of the samples were analyzed using four different metrics: Spearman correlation, Pearson correlation, Bray-Curtis dissimilarity, and Kullback-Leibler divergence. The network includes relationships that are significant (*P* < 0.05) for at least two of the above methods.

For each infant, we adjoined the genus abundance measurements for the top 15 genera from the NS and NPA samples and created a composite microbiota profile. Based on this composite data, we determined common patterns of genus abundance within individuals when comparing NS and NPA sites. We then used the composite data and Bray-Curtis dissimilarity metric to cluster subjects by partitioning around medoids (PAM). The optimal number of clusters was determined using the gap statistic and average silhouette width [35, 36].

### Replication analysis

To replicate the findings comparing NPA microbiota to bronchiolitis severity outcomes using the NS microbiota data [21], the NS samples were clustered separately using the weighted UniFrac distance and PAM clustering. The optimal number of clusters was determined by the average silhouette width. We then determined associations between the NS microbiota profiles and clinical covariates using chi-squared and Kruskal-Wallis tests as appropriate. Parallel to our previous analysis, a fixed-effects logistic regression model and a mixed-effects logistic regression model adjusting for 11 clinical variables were constructed using the NS clusters for each of the severity outcomes [21] (i.e., intensive care use [i.e., admission to intensive care unit and/or use of continuous positive airway pressure and/or intubation during inpatient stay, regardless of location] and hospital length of stay). For the intensive care use outcome, these models were then repeated on isolated microbiota profiles using membership in the profile of interest (e.g., *Haemophilus*-dominant profile versus all other subjects) as the independent variable.

## Results

Out of 921 subjects in the MARC-35 longitudinal cohort, site teams collected one NS and one NPA from 920 of these infants. We obtained optimal reads for 819 NS samples and 914 NPA samples. There were 815 subjects with optimal reads of both NS and NPA samples, and this group comprised the analytic cohort. Within this cohort, the median age at hospitalization was 3 months (IQR 2–6 months), the median weight at hospitalization was 6 kg (IQR 5–8 kg), and 31% of the infants had used antibiotics prior to hospitalization.

**Fig. 1:**
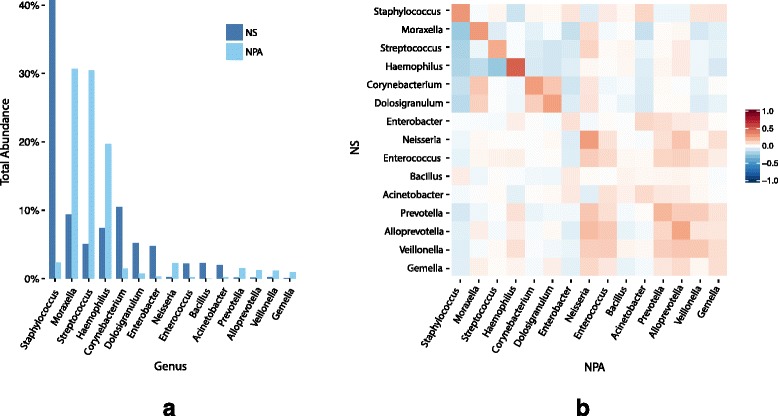
Comparison of nasal swab and nasopharyngeal microbiota. The genera abundances for the nasal swab and nasopharyngeal samples were calculated by taking the sum of all samples over each genus for each sample type. Combining the top 10 genera for each sample type gave 15 unique overall top genera. **a** The abundances of each of the top genera were calculated for both of the sample types. **b** For the top 15 genera, the Spearman correlations between the two anatomic sites are shown. The correlation heat map exhibits asymmetric behavior because it is comparing between the two sample types

We found 15 unique genera for comparative analysis after examining the top 10 abundant genera from the NS and NPA (Fig. 1a). The NS microbiota was dominated by the *Staphylococcus* genus (40.8%). The next most abundant genera were *Corynebacterium* (10.4%), *Moraxella* (9.3%), *Haemophilus* (7.4%), *Dolosigranulum* (5.2%), *Streptococcus* (5.0%), and *Enterobacter* (4.7%), which together with *Staphylococcus* accounted for 80% of the NS microbiota. The NPA microbiota was dominated by *Moraxella* (30.7%), *Streptococcus* (30.5%), and *Haemophilus* (19.7%) genera, which comprised over 80% of the microbiota. The abundances of the dominant genera in both sample types show high variability (Table 1). Additionally, the NS microbiota showed less bacterial richness and lower Shannon diversity index scores than the NPA microbiota, corresponding to the predominance of *Staphylococcus* in the NS samples. However, there were several NS (6.3%) and NPA (0.5%) samples that were completely dominated by one genus, indicating that this low diversity state may be a feature of a small percentage of infant upper airway microbiota at the time of hospitalization.

**Table 1:** Richness, alpha-diversity, and abundance by microbiota sample type

	Nasal swab		Nasopharyngeal aspirate	
Richness, median (IQR)				
Number of genera	6 (3–15)		12 (6.5–20)	
Alpha-diversity, median (IQR)				
Shannon index	0.58 (0.09–1.11)		0.90 (0.52–1.40)	
Relative abundance of 15 most common genera, mean (SD), maximum				
*Staphylococcus*	0.41 (0.43)	1.00	0.02 (0.10)	1.00
*Moraxella*	0.09 (0.21)	0.99	0.31 (0.34)	1.00
*Streptococcus*	0.05 (0.14)	0.98	0.30 (0.30)	1.00
*Haemophilus*	0.07 (0.20)	1.00	0.20 (0.31)	1.00
*Corynebacterium*	0.10 (0.22)	1.00	0.01 (0.06)	0.95
*Dolosigranulum*	0.05 (0.14)	1.00	0.01 (0.04	0.70
*Enterobacter*	0.05 (0.18)	1.00	0.00 (0.03)	0.48
*Neisseria*	0.00 (0.01)	0.19	0.02 (0.07)	0.75
*Enterococcus*	0.02 (0.12)	1.00	0.00 (0.01)	0.23
*Bacillus*	0.02 (0.14)	1.00	0.00 (0.00)	0.02
*Acinetobacter*	0.02 (0.12)	1.00	0.00 (0.02)	0.25
*Prevotella*	0.00 (0.01)	0.29	0.02 (0.05)	0.58
*Alloprevotella*	0.00 (0.01)	0.18	0.01 (0.04)	0.56
*Veillonella*	0.00 (0.01)	0.10	0.01 (0.03)	0.27
*Gemella*	0.00 (0.00)	0.06	0.01 (0.03)	0.52

Comparison of the richness, alpha-diversity (Shannon index), and genus abundances for the nasal swab and nasopharyngeal aspirate samples. The most common genera were determined by the union of the 10 most abundant genera from each sample type and are listed in order from most to least abundant

### Correlations between NS and NPA microbiota

**Fig. 2:**
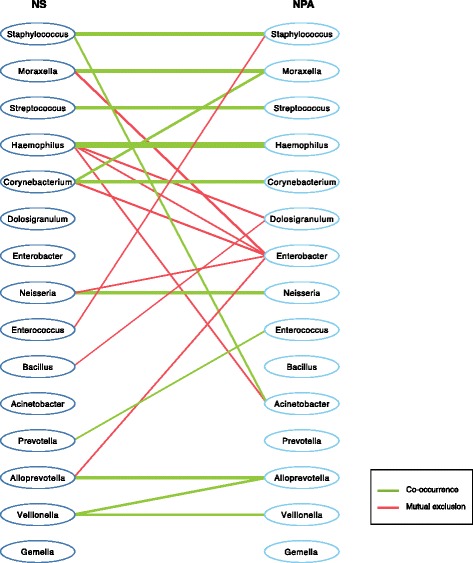
Network of significant intra-individual co-occurrence and co-exclusion associations between nasal swab and nasopharyngeal microbiota. The co-occurrence (green) and co-exclusion (red) relationships between the nasal swab (left) and nasopharyngeal (right) microbiota were assessed using four different methods: Spearman correlation, Pearson correlation, Bray-Curtis dissimilarity, and Kullback-Leibler divergence. To prevent the potential overestimation of associations that occur with the individual metrics, we retained in the network only associations between the top 15 genera that were found significant (*P* < 0.05) via bootstrapping for at least two of the aforementioned methods. Edges are weighted by the Spearman correlation value, with thicker lines indicating a larger correlation

A permutation test demonstrated that the within-individual mean correlation of 0.36 between the two upper airway sites while low was significantly higher than the randomly paired sample distribution (10,000 permutations, *P* = 0), which had a mean of 0.28 (SD = 0.004). The Spearman correlations between the top 15 genera from the NS and NPA are shown in Fig. 1b. *Haemophilus* has the greatest correlation between anatomic sites (*ρ* = 0.50). Additionally, there is a negative correlation between all of the top genera in NS (except *Bacillus*) and *Staphylococcus* in NPA, further illustrating the differences in *Staphylococcus* abundance between the NS and NPA samples. The between-site correlation network demonstrates that the top five genera in the NS and NPA co-occur within individuals, including *Haemophilus* and *Moraxella* (Fig. 2). Although *Staphylococcus* dominates the NS, the correlation network shows that *Staphylococcus* in NS is only significantly correlated with *Staphylococcus* and *Acinetobacter* in NPA.

**Fig. 3:**
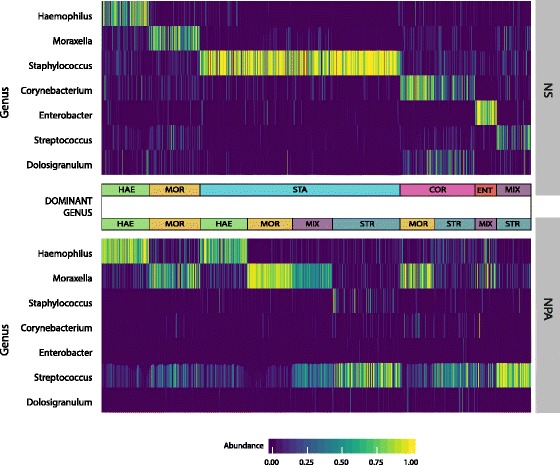
Clustering and composition of composite microbiota. For each subject in the study, adjoining the abundances of the top genera from the nasal swab and nasopharyngeal aspirate microbiotas created a composite microbiota sample. The Bray-Curtis dissimilarity was computed between each composite sample, and partitioning around medoids clustering was performed on the resulting dissimilarity values using 10 clusters. The heat map displays the abundances of the top seven genera for each of the resulting clusters, revealing common patterns of genus dominance between the nasal swab and nasopharyngeal microbiota. HAE, *Haemophilus*; MOR, *Moraxella*; STA, *Staphylococcus*; COR, *Corynebacterium*; ENT, *Enterobacter*; MIX, multiple genera; STR, *Streptococcus*

Clustering the composite samples generated by adjoining the NS and NPA genus abundances for each individual demonstrated common within individual patterns between the dominant genera in the NS and NPA samples (Fig. 3). Of particular interest are the first two clusters, which show that infants with *Haemophilus*- and *Moraxella*-dominant NS samples also have *Haemophilus*- and *Moraxella*-dominant NPA samples. Additionally, the NS clusters with a high abundance of *Staphylococcus* correspond to high abundances of the three major genera in the NPA (i.e., *Haemophilus*, *Moraxella*, *Streptococcus*), indicating that high *Staphylococcus* samples from NS map to all of the previously identified NPA microbiota profile groups discussed below [21].

### Nasal swab microbiota profiles

**Fig. 4:**
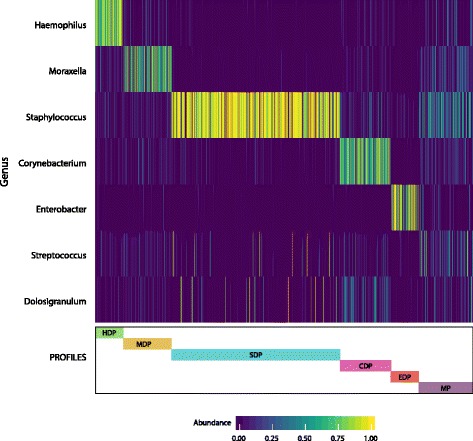
Composition of nasal swab microbiota profiles. Partitioning around medoids was performed on the weighted UniFrac distances between only the nasal swab genus abundances (independent of the nasopharyngeal aspirate genus abundances) using six clusters. The heat map shows the abundance of the top seven genera for each cluster. HDP, *Haemophilus*-dominant profile; MDP, *Moraxella*-dominant profile; SDP, *Staphylococcus*-dominant profile; CDP, *Corynebacterium*-dominant profile; EDP, *Enterobacter*-dominant profile; MP, mixed profile

Using PAM clustering, we previously reported four microbiota profiles generated from the NPA samples from infants with bronchiolitis: *Haemophilus*-dominant, *Moraxella*-dominant, *Streptococcus*-dominant, and mixed profiles [21]. In contrast to these four NPA microbiota profiles, PAM clustering of the NS samples generated six profiles: *Haemophilus*-dominant (7.2%), *Moraxella*-dominant (13.0%), *Staphylococcus*-dominant (44.5%), *Corynebacterium*-dominant (13.4%), *Enterobacter*-dominant (7.5%), and mixed (14.4%) profiles (Fig. 4). This PAM analysis of the NS gives a very large and well-defined cluster of subjects with a large abundance of *Staphylococcus* genus. Indeed, the *Staphylococcus*-dominant profile consists of 78% *Staphylococcus* and thus shows low bacterial richness and evenness (Table 2). Moreover, the infants in the *Staphylococcus*-dominant profile were younger compared to other profile groups (*P* < 0.001), with 85% of the infants in this profile being less than 6 months of age (Table 3). The infants in the NS *Haemophilus*-dominant profile were older (median 5 months; IQR 3–8 months), had higher weight (median 7 kg; IQR 6–8 kg), and were more likely to have used antibiotics prior to hospitalization (50.8%) as compared to the other profiles (all *P* < 0.001) (Table 3), results which are all similar to the NPA findings [21].

**Table 2:** Richness, alpha-diversity, and abundance by nasal swab microbiota profile

	*Haemophilus*-dominant profile, *n* = 59 (7.2%)	*Moraxella*-dominant profile, *n* = 106 (13.0%)	*Staphylococcus*-dominant profile, *n* = 363 (44.5%)	*Corynebacterium*-dominant profile, *n* = 109 (13.4%)	*Enterobacter*-dominant profile, *n* = 61 (7.5%)	Mixed profile, *n* = 117 (14.4%)
Richness, median (IQR)						
Number of genera	11 (8–9)	15 (5–23)	4 (2–7)	8 (5–12)	4 (3–6)	18 (10–27)
Alpha-diversity, median (IQR)						
Shannon index	0.77 (0.37–1.15)	1.03 (0.50–1.39)	0.11 (0.01–0.48)	0.89 (0.53–1.15)	0.37 (0.07–0.70)	1.44 (1.11–1.80)
Relative abundance of 10 most abundant genera, mean (SD)						
*Staphylococcus*	0.05 (0.08)	0.04 (0.07)	0.78 (0.35)	0.07 (0.13)	0.03 (0.06)	0.29 (0.24)
*Corynebacterium*	0.02 (0.03)	0.05 (0.08)	0.02 (0.05)	0.59 (0.27)	0.00 (0.01)	0.07 (0.09)
*Moraxella*	0.06 (0.09)	0.48 (0.33)	0.01 (0.02)	0.03 (0.06)	0.01 (0.03)	0.13 (0.13)
*Haemophilus*	0.74 (0.20)	0.04 (0.09)	0.00 (0.02)	0.03 (0.07)	0.01 (0.03)	0.06 (0.12)
*Dolosigranulum*	0.02 (0.04)	0.04 (0.07)	0.04 (0.17)	0.13 (0.17)	0.01 (0.03)	0.07 (0.14)
*Streptococcus*	0.03 (0.06)	0.05 (0.08)	0.04 (0.14)	0.04 (0.11)	0.01 (0.02)	0.14 (0.21)
*Enterobacter*	0.01 (0.06)	0.01 (0.04)	0.00 (0.02)	0.00 (0.01)	0.52 (0.41)	0.03 (0.09)
*Bacillus*	0.00 (0.01)	0.00 (0.00)	0.05 (0.20)	0.00 (0.00)	0.00 (0.00)	0.01 (0.08)
*Enterococcus*	0.00 (0.02)	0.01 (0.02)	0.04 (0.17)	0.01 (0.05)	0.01 (0.05)	0.01 (0.06)
*Acinetobacter*	0.00 (0.00)	0.12 (0.30)	0.00 (0.01)	0.00 (0.00)	0.02 (0.09)	0.02 (0.08)

Summary of the richness, alpha-diversity (Shannon index), and relative abundances of the most common genera for each of the nasal swab microbiota profiles as determined by partitioning around the medoid (PAM) clustering. The 10 most abundant genera were determined by taking the sum of the abundances for each genus over all the samples and are listed in order from most to least abundant

**Table 3:** Characteristics and clinical presentation of infants hospitalized for bronchiolitis by nasal swab microbiota profile

Variables	*Corynebacterium*-dominant profile, *n* = 109 (13.4%)	*Haemophilus*-dominant profile, *n* = 59 (7.2%)	*Moraxella*-dominant profile, *n* = 106 (13.0%)	*Enterobacter*-dominant profile, *n* = 61 (7.5%)	*Staphylococcus*-dominant profile, *n* = 363 (44.5%)	Mixed profile, *n* = 117 (14.4%)	*P* value
Characteristics							
Age (months), median (IQR)	3 (1–6)	5 (3–8)	4 (2–7)	4 (2–6)	3 (2–4)	4 (2–7)	**< 0.001**
< 2	36 (33.0)	7 (11.9)	24 (22.6)	14 (23.0)	132 (36.4)	23 (19.7)	**< 0.001**
2–5.9	44 (40.4)	25 (42.4)	48 (45.3)	34 (55.7)	178 (49.0)	58 (49.6)	
6–11.9	29 (26.6)	27 (45.8)	34 (32.1)	13 (21.3)	53 (14.6)	36 (30.8)	
Male sex	59 (54.1)	36 (61.0)	58 (54.7)	37 (60.7)	230 (63.4)	68 (58.1)	0.45
Race/ethnicity							0.07
Non-Hispanic white	53 (48.6)	23 (39.0)	49 (46.2)	20 (32.8)	177 (48.8)	38 (32.5)	
Non-Hispanic black	18 (16.5)	11 (18.6)	22 (20.8)	22 (36.1)	80 (22.0)	30 (25.6)	
Hispanic	34 (31.2)	24 (40.7)	31 (29.2)	17 (27.9)	93 (25.6)	44 (37.6)	
Other	4 (3.7)	1 (1.7)	4 (3.8)	2 (3.3)	13 (3.6)	5 (4.3)	
Parental history of asthma	30 (27.5)	15 (25.4)	42 (39.6)	26 (42.6)	124 (34.2)	37 (31.6)	0.18
Maternal smoking during pregnancy	16 (14.7)	4 (6.8)	14 (13.2)	14 (23.0)	50 (13.8)	13 (11.1)	0.19
Mode of birth							0.84
Vaginal birth	71 (65.1)	37 (62.7)	65 (61.3)	41 (67.2)	248 (68.3)	75 (64.1)	
C-section	38 (34.9)	21 (35.6)	38 (35.8)	20 (32.8)	111 (30.6)	41 (35.0)	
Prematurity (32–37 weeks)	17 (15.6)	12 (20.3)	15 (14.2)	12 (19.7)	60 (16.5)	22 (18.8)	0.87
Previous breathing problems before the index hospitalization*	23 (21.1)	12 (20.3)	27 (25.5)	17 (27.9)	62 (17.1)	26 (22.2)	0.26
History of eczema	17 (15.6)	12 (20.3)	20 (18.9)	13 (21.3)	61 (16.8)	21 (17.9)	0.92
Ever attended daycare	23 (21.1)	17 (28.8)	33 (31.1)	15 (24.6)	75 (20.7)	27 (23.1)	0.27
Aeroallergen sensitization†	0 (0.0)	1 (1.7)	2 (1.9)	0 (0.0)	7 (1.9)	2 (1.7)	0.68
Food sensitization†	21 (19.3)	9 (15.3)	22 (20.8)	13 (21.3)	64 (17.6)	23 (19.7)	0.92
Children at home	84 (77.1)	48 (81.4)	88 (83.0)	49 (80.3)	285 (78.5)	92 (78.6)	0.90
Mostly breastfed for the first 3 months of age	59 (54.1)	31 (52.5)	51 (48.1)	23 (37.7)	152 (41.9)	45 (38.5)	0.21
Smoke exposure at home	17 (15.6)	5 (8.5)	18 (17.0)	14 (23.0)	55 (15.2)	18 (15.4)	0.42
Antibiotic use before index hospitalization	29 (26.6)	30 (50.8)	44 (41.5)	27 (44.3)	96 (26.4)	28 (23.9)	**< 0.001**
Corticosteroid use before index hospitalization	13 (11.9)	13 (22.0)	19 (17.9)	11 (18.0)	46 (12.7)	19 (16.2)	0.31
Clinical presentation							
Month of hospitalization							0.06
November	10 (9.2)	3 (5.1)	10 (9.4)	10 (16.4)	32 (8.8)	5 (4.3)	
December	21 (19.3)	7 (11.9)	16 (15.1)	6 (9.8)	74 (20.4)	22 (18.8)	
January	31 (28.4)	24 (40.7)	32 (30.2)	15 (24.6)	97 (26.7)	36 (30.8)	
February	31 (28.4)	14 (23.7)	24 (22.6)	12 (19.7)	85 (23.4)	32 (27.4)	
March	12 (11.0)	8 (13.6)	21 (19.8)	14 (23.0)	40 (11.0)	15 (12.8)	
April	4 (3.7)	3 (5.1)	3 (2.8)	4 (6.6)	35 (9.6)	7 (6.0)	
Breathing problem began < 1 day before the index hospitalization	5 (4.6)	2 (3.4)	8 (7.5)	3 (4.9)	20 (5.5)	10 (8.5)	0.67
Weight at presentation (kg), median (IQR)	6 (5–8)	7 (6–8)	6 (5–8)	6 (5–8)	6 (5–7)	7 (5–8)	**< 0.001**
Respiratory rate at presentation (per minute), median (IQR)	48 (40–59)	50 (40–60)	49 (42–60)	50 (42–64)	50 (40–60)	50 (42–60)	0.26
Oxygen saturation at presentation							**0.006**
< 90%	7 (6.4)	13 (22.0)	4 (3.8)	7 (11.5)	31 (8.5)	11 (9.4)	
90–93%	23 (21.1)	9 (15.3)	14 (13.2)	5 (8.2)	50 (13.8)	16 (13.7)	
≥ 94%	74 (67.9)	35 (59.3)	88 (83.0)	46 (75.4)	274 (75.5)	90 (76.9)	
Retractions on examination							0.32
None	18 (16.5)	6 (10.2)	24 (22.6)	13 (21.3)	58 (16.0)	17 (14.5)	
Mild	42 (38.5)	21 (35.6)	45 (42.5)	23 (37.7)	160 (44.1)	55 (47.0)	
Moderate/severe	43 (39.4)	30 (50.8)	33 (31.1)	23 (37.7)	127 (35.0)	45 (38.5)	
Wheezing on examination	63 (57.8)	30 (50.8)	65 (61.3)	37 (60.7)	214 (59.0)	77 (65.8)	0.31
Received antibiotics during pre-hospitalization visit	28 (25.7)	34 (57.6)	35 (33.0)	25 (41.0)	104 (28.7)	41 (35.0)	**< 0.001**
Received corticosteroids during pre-hospitalization visit	6 (5.5)	5 (8.5)	5 (4.7)	6 (9.8)	34 (9.4)	17 (14.5)	0.15
Virology							0.11
Sole RSV infection	56 (51.4)	30 (50.8)	69 (65.1)	34 (55.7)	225 (62.0)	60 (51.3)	
Sole rhinovirus infection	7 (6.4)	4 (6.8)	4 (3.8)	8 (13.1)	17 (4.7)	7 (6.0)	
RSV + rhinovirus coinfection	14 (12.8)	3 (5.1)	14 (13.2)	7 (11.5)	44 (12.1)	15 (12.8)	
RSV + non-rhinovirus pathogens	14 (12.8)	15 (25.4)	9 (8.5)	6 (9.8)	34 (9.4)	15 (12.8)	
Rhinovirus + non-RSV pathogens	2 (1.8)	2 (3.4)	3 (2.8)	2 (3.3)	9 (2.5)	5 (4.3)	
Neither RSV nor rhinovirus‡	11 (10.1)	5 (8.5)	6 (5.7)	4 (6.6)	26 (7.2)	9 (7.7)	
No viral pathogens	5 (4.6)	0 (0.0)	1 (0.9)	0 (0.0)	8 (2.2)	6 (5.1)	
Viral genomic load (CT-value), median IQR							
RSV	22 (20–25)	22 (21–26)	22 (21–25)	23 (21–25)	23 (21–25)	22 (21–27)	0.63
RV	28 (26–31)	29 (24–34)	28 (27–36)	27 (25–34)	31 (27–36)	30 (27–36)	0.60
Outcomes							
Intensive care use‡	20 (18%)	16 (27%)	6 (6%)	13 (21%)	47 (13%)	21 (18%)	
Hospital length of stay ≥ 5 days	10 (9%)	19 (32%)	16 (15%)	8 (13%)	47 (13%)	18 (15%)	
Hospital length of stay (day), median (IQR)	2 (1–4)	2 (2–5)	2 (1–3)	2 (1–3)	2 (1–3)	2 (1–3)	

Data are number (%) of infants unless otherwise indicated. Percentages may not be equal to 100, because of missingness

Patient characteristics and hospital course were compared using chi-square test or Kruskal-Wallis test across the identified nasopharyngeal microbiota profiles

*CT* cycle threshold, *IQR* interquartile range, *RSV* respiratory syncytial virus

*Defined as an infant having cough that wakes him/her at night and/or causes emesis, or when the child has wheezing or shortness of breath without cough

†Defined by having one or more positive values for allergen-specific IgE

‡Defined as admission to intensive care unit and/or use of mechanical ventilation (continuous positive airway pressure and/or intubation during inpatient stay, regardless of location) at any time during the index hospitalization

### Nasal swab microbiota profiles and Bronchiolitis severity

Using the NPA data, our group previously reported that infants with a *Haemophilus*-dominant NPA microbial profile had increased odds of intensive care use and risk of hospital length of stay of three or more days when compared to those with a *Moraxella*-dominant microbiota profile [21]. In the current analysis, we were able to replicate these severity outcome associations in NS microbiota profiles, finding that subjects with the *Haemophilus*-dominant NS profile were more likely to have intensive care use (unadjusted OR 6.20, *P* < 0.001; adjusted OR 6.43, *P* < 0.001) and a hospital length of stay of five or more days (unadjusted OR 3.57, *P* = 0.004; adjusted OR 4.31, *P* = 0.002) (Table 4).

**Table 4:** Unadjusted and multivariate associations of nasal swab microbiota profiles with bronchiolitis severity outcomes

	Unadjusted models		Adjusted models*	
Severity outcomes	OR (95% CI)	*P* value	OR (95% CI)	*P* value
1) Intensive care use				
All profiles				
*Haemophilus*-dominant profile	*6.20* (*2.38*–*18.28*)	*< 0.001*	*6.43* (*2.25–20.51*)	< *0.001*
*Moraxella*-dominant profile	Reference		Reference	
*Staphylococcus*-dominant profile	2.54 (1.14–6.78)	0.04	2.17 (0.93–5.98)	0.10
*Corynebacterium*-dominant profile	3.75 (1.52–10.62)	0.007	4.15 (1.59–12.37)	0.005
*Enterobacter*-dominant profile	4.51 (1.68–13.53)	0.004	4.84 (1.67–15.46)	0.004
Mixed profile	3.86 (1.59–10.85)	0.005	3.37 (1.29–9.99)	0.02
*Haemophilus* vs. all others				
Combined non-*Haemophilus* profiles	Reference		Reference	
*Haemophilus*-dominant profile	*2.21* (*1.17*–*3.99*)	*0.01*	*2.48* (*1.19*–*5.03*)	*0.01*
*Moraxella* vs. All others				
Combined non-*Moraxella* profiles	Reference		Reference	
*Moraxella*-dominant profile	*0.30* (*0.11*–*0.64*)	*0.01*	*0.32* (*0.12*–*0.72)*	*0.01*
2) Hospital length of stay ≥ 5 days				
All profiles				
*Haemophilus*-dominant profile	*3.57* (*1.52*–*8.77*)	*0.004*	*4.31* (*1.73*–*11.11*)	*0.002*
*Moraxella*-dominant profile	Reference		Reference	
*Staphylococcus*-dominant profile	1.43 (0.72–3.09)	0.33	1.24 (0.61–2.75)	0.57
*Corynebacterium*-dominant profile	2.03 (0.91–4.76)	0.09	2.02 (0.86–4.95)	0.11
*Enterobacter*-dominant profile	1.45 (0.52–3.89)	0.46	1.37 (0.48–3.83)	0.55
Mixed profile	1.75 (0.78–4.11)	0.18	1.72 (0.73–4.20)	0.22

Significant results of interest are in italics

Patient level variables include age at hospitalization, sex, race/ethnicity, gestational age, number of previous breathing problems (i.e., infant having cough that wakes him/her at night and/or causes emesis or when the child has wheezing or shortness of breath without cough), daycare attendance, presence of other children living in home, history of antibiotic use (i.e., infant has taken antibiotics at any time prior to hospitalization), history of corticosteroid use (i.e., infant has taken corticosteroids, inhaled or systemic, at any time prior to hospitalization), use of antibiotics during the pre-hospitalization visit (i.e., infant received antibiotics during pre-admission), and respiratory viruses detected by PCR

*CI*, confidence interval, *OR*, odds ratio

*Mixed-effects logistic regression model adjusting for 11 patient-level variables with collection site as a random effect

Analysis of the NS microbiota profiles found higher odds ratios than the NPA microbiota profiles for the severity outcomes. Isolating the *Haemophilus* and *Moraxella* NS profiles showed increased odds of intensive care use for the *Haemophilus*-dominant profile and a protective association of the *Moraxella*-dominant profile (Table 4), coincident with the patterns observed in NPA profiles of these samples. While the NS microbiota profiles did not retain the significant association with a hospital stay of three or more days, they were significantly associated with a length of stay of five or more days. Thus, the association between the *Haemophilus*-dominant profile and a longer hospital length of stay, as well as the protective nature of *Moraxella* for the hospital length of stay outcome, was maintained in the NS data.

## Discussion

In this multicenter study of 815 infants with both NS and NPA samples, we found within-individual correlations between dominant genera in NS and NPA microbiota. However, the NS and NPA samples also revealed distinct compositions, with an increased abundance of *Staphylococcus* in the NS microbiota. Using an independent analysis of the NS microbiota, we were able to replicate a previously published association between NPA microbiota and bronchiolitis severity outcomes [21] despite sample processing differences. Our findings indicate that the associations of *Haemophilus*-dominant and *Moraxella*-dominant profiles with clinical outcomes should be consistent between NS and NPA sample types.

There was a modest but statistically significant within-individual relationship between the bacterial microbiota of the two upper airway sites. Further analysis of these correlations revealed common mappings between NS and NPA genus abundances, particularly the *Haemophilus*-dominant and *Moraxella*-dominant NS samples that were maintained in the NPA microenvironment. Beyond these two genera, the mapping between NS and NPA demonstrated inconsistency between the sample types among the dominant genera. For example, the majority of infants with dominant *Staphylococcus* in the NS samples had NPA samples that were dominated by *Haemophilus*, *Moraxella*, or *Streptococcus*. Thus, NS samples with a high abundance of *Staphylococcus* alone may not be informative for the corresponding most abundant genera in the NPA. This is further supported by the correlation network (Fig. 2), which shows that *Staphylococcus* in NS only co-occurs with *Staphylococcus* and *Acinetobacter* in NPA. We also found that some samples—particularly NS samples dominated by *Staphylococcus*—were almost entirely composed of a single genus. Regardless of the dominance of *Staphylococcus* in NS samples, the large study size allowed ample data for reliable analysis. Despite having compositional differences, the correlations between the NS and NPA microbiota confirm that NSs are a useful clinical sample type. However, researchers should be cautious of the complications imposed by the abundance of *Staphylococcus* in the NS samples.

Almost half of the MARC-35 infants had a *Staphylococcus*-dominant profile with relatively low bacterial diversity. The difference in bacterial richness and diversity between the *Staphylococcus*-dominant profile and the other NS profiles raises the question of whether the high abundance of *Staphylococcus*, commonly found in the anterior nares [20, 37–39], is associated with clinical outcomes. Although *Staphylococcus* is often found in the anterior nares of healthy patients [20, 39], studies have found a higher abundance of *Staphylococcus* in the anterior nares of patients admitted to the intensive care unit [38] and adults with chronic rhinosinusitis [37], which may imply a difference in NS *Staphylococcus* abundance between sick and healthy individuals. Of more relevance to infants with bronchiolitis, *Staphylococcus aureus* has not only been shown to enhance the replication and infectivity of rhinovirus [40] and influenza [41], but also a *Staphylococcus*-dominant airway microbiota is associated with an increased likelihood of severe bronchiolitis [42]. However, because the 16S rRNA gene sequencing approach is insufficient to reliably resolve data at the species level, it is unclear whether the *Staphylococcus* OTU in our data is in fact *Staphylococcus aureus* or another species of *Staphylococcus*. While the species of *Staphyloccocus* in our data may determine how it impacts other dominant genera, the presence of *Staphylococcus* nonetheless has potential relevance for respiratory outcomes.

In the present analysis, we have demonstrated the usefulness of this easily obtained sample type when compared with NPA samples. Specifically, we were able to recapitulate the relationship between *Haemophilus*-dominant nasal microbiota and bronchiolitis severity [21], as well as the protective nature of *Moraxella* in the airway microbiota [21, 43], with NS samples among infants with bronchiolitis (Table 4). Furthermore, the NS microbiota profiles preserved associations with age, weight, and antibiotics use (Table 3). We conclude that for associations with *Haemophilus* and *Moraxella*, NS and NPA provide similar results. However, because many *Haemophilus* and *Moraxella* dominant samples in NPA were dominated by *Staphylococcus* in the NS samples for the same individuals, the ability to predict bronchiolitis severity outcomes may be more limited for *Staphylococcus*-dominant NS samples. Additionally, the associations between other microbiota dominant profiles and clinical outcomes may differ between the anterior nares and nasopharyngeal microenvironments.

Our study has some potential limitations. First, variations between the NS and NPA microbiota may have been amplified due to differences in NS and NPA sample handling (e.g., temperature at which initially stored). Studies of fecal microbiota have found that while variations in transport media can significantly alter the microbial composition of microbiota samples, differences in storage temperature should have a relatively little effect on the resulting microbial composition [44, 45]. Although immediately freezing the NS samples would have allowed a direct comparison to the NPA samples, the NS samples were collected and processed in a manner easily replicable in outpatient clinics or in homes for community-based studies [46–50]. Mailing NS samples has been used for viral detection [47–49], but this sample collection technique has not previously been used to examine the microbiota even though both viruses, and the microbiota are associated with acute bronchiolitis severity outcomes [8, 43, 51] as well as other respiratory outcomes [41]. Although viral detection is improved when nasal samples are collected from deeper within the nasopharynx than from the anterior nares [52], the more easily accessible NS samples have been useful for detecting viruses in community studies [53]. Allowing participants to provide a mailed NS sample would remove obstacles typically faced by researchers who want to examine both viral and microbial exposures from community-based samples. In this study, despite the differences in initial storage temperature of the samples, we were able to use the NS sample data to replicate the previous association between NPA microbiota and bronchiolitis severity.

In addition, as the samples in our study were obtained in the context of acute bronchiolitis hospitalization, the within-individual correspondence we observed may not be reproducible in healthy infants. However, our study demonstrates that NS microbiota data is relevant in the context of acute illness and is associated both with the microbial composition of the nasopharynx as well as with clinically relevant outcomes. Another potential limitation is that we did not address the dynamics of the microbiota at these sites. The airway microbiota changes over time via environmental exposure and the natural progression of microbial composition in the airway [8, 43, 54]. Although our findings show the associations in infant nasal microbiota, the relationship between anterior nare samples and nasopharyngeal samples in older children is unknown. Our data should facilitate further investigations into this important domain.

## Conclusions

In this multicenter cohort study of infants hospitalized with bronchiolitis, we found modest but statistically significant intra-individual correlations between NS and NPA microbiota, especially for *Haemophilus* and *Moraxella*. Given the overlap of the microbiota structure between the sample types, we were able to use NS data to replicate the previously identified associations between NPA microbiota and severity of illness (as measured by intensive care use and hospital length of stay). Our investigation also reveals important differences between NS and NPA samples—particularly the high abundance of *Staphylococcus* in the NS. However, despite the variations between the NS and NPA microbiota and the differential handling of the specimens, the considerable overlap of the microbiota between the anatomic sites indicates that NS can provide robust and useful samples in young children.

## Additional file

Additional file 1: R code for statistical analysis. (PDF 33 kb)

## Abbreviations

NPA: Nasopharyngeal aspirate
NS: Nasal swab
OR: Odds ratio
OTU: Operational taxonomic unit
PAM: Partitioning around medoid
SD: Standard deviation
