# Supplementary material for: The association between anterior nares and nasopharyngeal microbiota in infants hospitalized for bronchiolitis
Source: Microbiome. 2018 Jan 3;6:2. doi: 10.1186/s40168-017-0385-0 (PMC5751828; doi:10.1186/s40168-017-0385-0)
Supplement: Additional file 1: — R code for statistical analysis. (PDF 33 kb) [file 40168_2017_385_MOESM1_ESM.pdf]

```
## Load libraries
library(reshape2)
library(ggplot2)
library(ggthemes)
library(scales)
library(vegan)
library(cluster)
library(viridis)

## Load data
meta <- read.csv('MS5_metadata.csv', # metadata file
           colClasses = c(rep('character', 4), 'numeric', rep('factor', 12)))
load('NPA-NS-genus.Rdata') # genus abundance table (subject x genus)
load('NPA-NS-wUF.Rdata') # weighted UniFrac matrix


### Plots

## Set up data
subj.ids <- meta$SubjectID
npa.ids <- meta$SampleID_NPA
ns.ids <- meta$SampleID_NS

top.10.npa <- names(sort(apply(genus[npa.ids, ], 2, sum), decreasing = TRUE)[1:10])
top.10.ns <- names(sort(apply(genus[ns.ids, ], 2, sum), decreasing = TRUE)[1:10])

top.genera <- unique(union(top.10.ns, top.10.npa))
top.genera <- names(sort(apply(genus[, top.genera], 2, sum), # order by overall abundance
                         decreasing = TRUE))

genus.top <- genus[, top.genera]
genus.top$SampleID <- rownames(genus.top)
genus.top$type <- factor(gsub("\\d", "", rownames(genus.top)), levels = c('NS', 'NPA'))

n <- dim(meta)[1]

col.scale <- colorRampPalette(rev(
  c(
    "#67001F",
    "#B2182B",
    "#D6604D",
    "#F4A582",
    "#FFFFFF",
    "#92C5DE",
    "#4393C3",
    "#2166AC",
    "#053061"
  )
))
col.pair <- ptol_pal()(6)

# Melt data
m.genus.top <- melt(genus.top, id.vars = c('SampleID', 'type'))
```

```
## Figure 1a: Bar plot
tgp <-
  ggplot(m.genus.top, aes(x = variable, fill = type, weight = value)) +
  geom_bar(aes(y = (..count..) / 815), position = "dodge", width = 0.7) +
  scale_y_continuous(labels = percent, expand = c(0, 0)) +
  theme_tufte(base_family = "Helvetica",
              base_size = 12,
              ticks = TRUE) +
  theme(axis.text.x = element_text(angle = 60, hjust = 1)) +
  theme(axis.line = element_line(colour = "grey", lineend = 400),
        axis.line.y = element_blank()) +
  labs(y = 'Total Abundance',
       x = 'Genus',
       title = NULL,
       fill = NULL) +
  scale_fill_manual(values = col.pair, breaks = c('NPA', 'NS')) +
  theme(legend.position = c(.8, .85))
tgp

## Figure 1b: Correlation plot
cormat <-
  cor(genus.top[ns.ids, top.genera], genus.top[npa.ids, top.genera], method =
        'spearman')
m.cormat <- melt(cormat)
gg.cor <- ggplot(data = m.cormat, aes(x = Var1, y = Var2, fill = value)) +
  geom_raster() +
  theme_tufte(base_family = "Helvetica", base_size = 12) +
  scale_fill_gradientn(colors = col.scale(100),
                       limit = c(-1, 1),
                       name = "") +
  scale_x_discrete(limits = top.genera) + scale_y_discrete(limits = rev(top.genera)) +
  labs(x = 'NPA', y = 'NS') +
  theme(axis.text.x = element_text(angle = 60, hjust = 1)) +
  theme(legend.key.size = unit(.5, 'cm'))
gg.cor


## Figure 3: Co-cluster Heatmap
genus.co <- cbind(genus.top[npa.ids, top.genera], genus.top[ns.ids, top.genera])
genus.co.bc <- vegdist(genus.co, method = 'bray') # calculate Bray-Curtis

k <- 10
clust.co <- pam(as.matrix(genus.co.bc), k = k, diss = TRUE)

genus.top$co.cluster <- rep(NA, dim(genus.top)[1])
genus.top[ns.ids, 'co.cluster'] <- clust.co$clustering
genus.top[npa.ids, 'co.cluster'] <- clust.co$clustering

# Plot heatmap
genus.top[c(ns.ids, npa.ids), 'SubjectID'] <- rep(subj.ids, 2) # match samples to subject
m.co.clust <-
  melt(genus.top[, c(top.genera[1:10], 'SubjectID', 'type', 'co.cluster')],
       id.vars = c('SubjectID', 'type', 'co.cluster'))
```

```
gg.co <-
  ggplot(m.co.clust, aes(
    x = reorder(SubjectID, co.cluster),
    y = variable,
    fill = as.numeric(value)
  )) +
  facet_grid(type ~ .) +
  geom_raster() +
  scale_fill_viridis(name = "Abundance") +
  labs(x = NULL, y = 'Genus', title = NULL) +
  theme_tufte(base_family = "Helvetica", base_size = 10) +
  theme(axis.ticks = element_blank()) +
  theme(axis.text.x = element_blank()) +
  theme(strip.background = element_rect(fill = 'grey', size = 1, color =
                                          'white')) +
  theme(legend.title = element_text(size = 8, vjust = 1)) +
  theme(legend.title.align = 1) +
  theme(legend.text = element_text(size = 6)) +
  theme(legend.position = "bottom") +
  theme(legend.key.size = unit(.75, "cm")) +
  theme(plot.margin = unit(c(1, 1, 0, 1), "cm"))
gg.co

## Figure 4: NS Cluster Heatmap
k <- 6
clust.ns <- pam(wUF[ns.ids, ns.ids], k = k, diss = TRUE)

genus.top.ns <- genus.top[ns.ids, ]
genus.top.ns$cluster <- clust.ns$clustering

# Plot heatmap
m.ns.clust <-
  melt(genus.top.ns[, c(top.10.ns[1:7], 'SampleID', 'cluster')],
       id.vars = c('SampleID', 'cluster'))

gg.ns <-
  ggplot(m.ns.clust, aes(
    x = reorder(SampleID, cluster),
    y = variable,
    fill = value
  )) +
  geom_raster() +
  scale_fill_viridis(name = "Abundance") +
  labs(x = NULL, y = 'Genus', title = NULL) +
  theme_tufte(base_family = "Helvetica", base_size = 10) +
  theme(axis.ticks = element_blank()) +
  theme(axis.text.x = element_blank()) +
  theme(strip.text = element_text(hjust = 0)) +
  theme(legend.title = element_text(size = 8, vjust = 1)) +
  theme(legend.title.align = 1) +
  theme(legend.text = element_text(size = 6)) +
  theme(legend.position = "bottom") +
  theme(legend.key.size = unit(.75, "cm"))
```

```
gg.ns


### Models
require(lme4)

## Intensive Treatment Outcome

# Set reference cluster
clust <- as.numeric(meta$cluster)
clust[which(clust == 3)] <- 0 # Moraxella-dominant cluster as reference
# clust <- as.numeric(clust == 2) # Haemophilus-dominant against all others
# clust <- as.numeric(clust == 3) # Moraxella-dominant against all others
meta$clust <- factor(clust)

# Unadjusted
m.it <-
  glm(IntensiveTreatment ~ clust,
      data = meta,
      family = binomial())
summary(m.it)
exp(cbind(OR = coef(m.it), confint(m.it)))

# Adjusted
m.it.adj <-
  glmer(
    IntensiveTreatment ~ clust + Age_Months + intake_sex + raceethn + gestage +
      prev_breathingprob + antibiotics_life + intake_daycare + children_in_home +
      intake_antibiotics + corticosteroids_life + NoPathogens_NPA + (1 | site),
    data = na.omit(meta),
    family = binomial(),
    glmerControl(optimizer = "bobyqa", optCtrl = list(maxfun = 100000))
  )
summary(m.it.adj)
exp(cbind(OR = attr(m.it.adj, 'beta')[1:6], confint(m.it.adj)))

## Length-of-stay outcome

# Unadjusted
m.los <- glm(LOS_5dys ~ clust, data = meta, family = binomial())
summary(m.los)
exp(cbind(OR = coef(m.los), confint(m.los))) # odds-ratios and CIs

# Adjusted
m.los.adj <-
  glmer(
    LOS_5dys ~ clust + Age_Months + intake_sex + raceethn + gestage + prev_breathingprob +
      antibiotics_life + intake_daycare + children_in_home + intake_antibiotics +
      corticosteroids_life + NoPathogens_NPA + (1 | site) ,
    data = na.omit(meta),
    family = binomial(),
    glmerControl(
      optimizer = "bobyqa",
      optCtrl = list(maxfun = 100000),
```

```
      check.conv.hess = .makeCC(action = "warning", tol = 1e-8),
      check.conv.grad = .makeCC("warning", tol = 1e-3, relTol = NULL)
    )
  )
summary(m.los.adj)
exp(cbind(OR = attr(m.los.adj, 'beta'), confint(m.los.adj))) # odds-ratios and CIs
```
